# A Specific Criteria-Based Guideline Improves Compliance With General Surgery Ambulatory Care Standards and Reduces Overcrowding in “Hot Clinic”: A Quality Improvement Study

**DOI:** 10.7759/cureus.31984

**Published:** 2022-11-28

**Authors:** Amal A Anwer, Jigar Shah, Shahin Hajibandeh, Moustafa Mansour, Shahab Hajibandeh

**Affiliations:** 1 Department of General Surgery, University Hospital of Wales, Cardiff, GBR; 2 Department of General Surgery, North Manchester General Hospital, Manchester, GBR; 3 Department of General surgery, Royal Stoke University Hospital, Stoke-on-Trent, GBR

**Keywords:** quality improvement, hot clinic, guideline, ambulatory care, general surgery

## Abstract

Background

A general surgery hot clinic is designed for the assessment and management of acute general surgical patients in ambulatory settings to avoid unnecessary hospital admissions. Overcrowding in the hot clinic is a major issue in many general surgical settings, and it is thought to be due to a lack of specific criteria-based guideline for identifying eligible patients for ambulatory care. We aimed to perform a prospective audit to assess what proportion of hot clinic patients meets the criteria for ambulatory care. Our second objective was to implement a specific criteria-based guideline and monitoring program to improve compliance with the ambulatory care criteria.

Methods

The audit included three cycles: baseline audit (30 days in September 2018), first re-audit (30 days in January 2019), and second re-audit (30 days in May 2019). During each cycle, all consecutive patients who attended the general surgery hot clinic were included. Compliance with the hot clinic standards was considered as the outcome measure. We considered compliance of 100% as a target for each standard. A specific criteria-based guideline for the hot clinic was implemented after the baseline audit. A monitoring program was designed to monitor and maintain compliance with the hot clinic guideline.

Results

During the baseline audit, 224 patients were seen in the general surgery hot clinic. After the implementation of the guideline, this was reduced to 40 patients during the first re-audit and 42 patients during the second re-audit. There was a significant difference in the median number of patients seen per day between the baseline audit and the first re-audit [(7 (6-8) vs 1 (1-2), P < 0.0001] and between the baseline audit and the second re-audit [(7 (6-8) vs 1 (1-2), P < 0.0001]. During the baseline audit, only 19% of patients were seen by the on-call general surgery team prior to a hot clinic; this improved to 100% in the first re-audit (P < 0.0001) and remained 100% in the second re-audit (P < 0.0001). During the baseline audit, only 19% of patients met the eligibility criteria for review in a hot clinic; this improved to 100% in the first re-audit (P < 0.0001) and remained 100% in the second re-audit (P < 0.0001).

Conclusions

A criteria-based hot clinic guideline suggested in this study improved compliance with general surgery ambulatory care standards, the efficiency of general surgery hot clinic, and overcrowding in general surgery hot clinic. A continuous monitoring program led by an on-call junior general surgery doctor helped to maintain the aforementioned improvements.

## Introduction

The number of emergency general surgery admissions has been increasing steadily over the past decades resulting in a significant strain on National Health Service (NHS) clinical and financial resources [1]. In order to reduce the costs to the healthcare provider and the inpatient burden to the acute surgical team, general surgery “hot clinic” has been introduced [2]. Hot clinic is designed for the assessment and management of acute general surgical patients in the ambulatory setting. It has been argued that up to 30% of acute general surgical patients could be managed via ambulatory pathways where unnecessary hospital admissions can be avoided [3].

Although general surgery hot clinics can help to improve patient flow in acute general surgical settings, the lack of specific criteria-based guidelines for identifying eligible patients for ambulatory care could have contrary effects. Overcrowding in hot clinics is a major issue in many general surgical settings, negatively affecting the efficiency of ambulatory care and patient satisfaction. Overcrowding mainly occurs for two reasons. First, due to the lack of specific criteria-based guidelines for including a patient in a hot clinic, many patients who do not meet the criteria for ambulatory care but can be managed in the outpatient settings are inappropriately booked in hot clinics. Second, the increasing number of daily referrals to emergency general surgery teams from the community, accident and emergency, and other specialties can easily tempt the on-call general surgery clinician to utilize the hot clinic as an excuse for not reviewing the referred patient on the same day as a referral and booking the patient in hot clinic. The second reason also contributes to the problem caused by the first reason making a vicious circle resulting in overcrowding in hot clinics.

Booking a patient in a hot clinic without reviewing the patient is not acceptable and contributes to including ineligible patients for review in a hot clinic; therefore, in order to decide whether a patient is eligible for ambulatory care, as an absolute requirement the patient must be reviewed clinically by the on-call general surgery team on the same day as a referral. Once the referred patient is reviewed, the on-call general surgery team should decide to investigate and manage the patient as an inpatient, ambulatory patient, or outpatient. The patient should be booked in the hot clinic as an ambulatory patient only for the following reasons: a review of already organized investigations for a patient who has already been seen; reassessment of a patient who has already been seen; and reassessment of a recently discharged inpatient.

We aimed to perform a prospective baseline audit to assess how many patients are seen in general surgery hot clinics on average and what proportion of hot clinic patients meet the criteria for ambulatory care. Our second objective was to implement a specific criteria-based guideline and monitor the program to improve compliance with the ambulatory care criteria.

## Materials and methods

This study has been reported in line with the Standards for QUality Improvement Reporting Excellence (SQUIRE) criteria. The protocol for the study is registered in the Chinese Clinical Trial Registry (Registration number: ChiCTR2000029369). Upon gaining approval from the Clinical Governance Development Unit, a prospective clinical audit was conducted in the general surgery department of a district general hospital located in the North West of England. As this was an audit, approval was not sought from the ethics committee. The audit included three cycles: baseline audit (30 days in September 2018), first re-audit (30 days in January 2019), and second re-audit (30 days in May 2019). During each cycle, all consecutive patients who attended the general surgery hot clinic were included. The audit loop was completed after the first re-audit; the second re-audit was performed to assess whether compliance is maintained.

Outcome measures and standards

We believe that the criteria specified in Table 1 would help to prevent overcrowding in the general surgery hot clinic. Compliance with the hot clinic standards was considered as the outcome measure. The standards for the audit are shown in Table 2. We considered compliance of 100% as the target for each standard.

**Table 1:** Criteria for reviewing a patient in general surgery hot clinic GP: general practitioner; A+E: Accident and emergency.

Absolute Requirement for a Patient to Be Seen in Hot Clinic
Patients must have been seen and assessed by the on-call team prior to being booked in a hot clinic regardless of the source and time of referral (GP, A+E, or other hospitals).
Who Can Be Seen in Hot Clinic?
Review of already organized investigations for a patient who has already been seen: This refers to patients who have already been seen by the on-call team, and review of the results of an already organized investigation (repeat blood tests, US scan, or CT scan) is required.
Reassessment of a patient who has already been seen: This refers to patients who have already been seen by the on-call team, and a second review is required to monitor the patient and decide whether admission is required.
Reassessment of recently discharged inpatients: This refers to patients who have recently been discharged, and a second review is required to monitor the patient (repeat blood tests, drain removal, etc.) or to decide whether readmission is required.
Who Should Not Be Seen in Hot Clinic?
Suspected biliary colic with resolved symptoms: Patients with suspected biliary colic with resolved symptoms should not be brought back to the hot clinic for an ultrasound (US) scan. US scan should be organized as outpatient, and the on-call registrar/senior house officer (SHO) who reviewed the patient must dictate a letter to the consultant on-call at the time of review to follow up on the results.
Rectal (PR) bleeding: Patients with PR bleeding should be seen by the on-call team, and decisions for admission or outpatient endoscopic assessment should be made at the time of review. The on-call registrar/SHO who reviewed the patient must dictate a letter to the consultant on-call at the time of review to follow up on the results.
Abscesses: Patients with abscesses must be seen by the on-call team on the same day as a referral to decide whether incision or drainage is required. In case incision or drainage is required, the patient should be booked and consented for incision and drainage and brought to Surgical Triage Unit (STU) as a theater case rather than a hot clinic case.

**Table 2:** The audit standards and the target for each standard SHO: Senior house officer.

No.	Standard	Target %
1	Had the patient been seen by the on-call registrar/SHO before the appointment?	100%
2	Did the patient meet either of the following criteria for hot clinic review?: a review of already organized investigations for a patient who has already been seen, reassessment of a patient who has already been seen, or reassessment of recently discharged inpatients.	100%

Data collection

For each patient, a data collection proforma was completed. The proforma included the following items: (1) “Had the patient been seen by the on-call team before the appointment?”; (2) “Did the patient meet the criteria for hot clinic review?”; (3) “What was the criterion for hot clinic review?”. Data collection was performed by the on-call general surgery junior clinician based at Surgical Triage Unit (STU) where the hot clinic was located. The clinical review documented in the medical notes by the general surgery team prior to the hot clinic appointment was considered as evidence to complete the proforma.

Implementation of criteria-based guideline for hot clinic

A specific criteria-based guideline for the hot clinics was developed (Table 1). A briefing session was organized for all general surgery clinicians, which involved clarification about the inclusion and exclusion criteria for hot clinic review and case-based discussion to practice the criteria. The online version of the guideline was provided to all clinicians, and hard copies of the guideline were displayed as wall posters in STU and the hot clinic. The clinicians were also informed about the monitoring program through which the lack of compliance with the guideline would be detected, and the corresponding clinician would be notified.

Sustaining improved compliance with hot clinic guideline

A monitoring program was designed to monitor and maintain compliance with the hot clinic guideline. The on-call general surgery junior clinician based at STU where the hot clinic was located was responsible to assess the clinical notes of the patients attending the hot clinic to ensure compliance with the guideline. In case a deviation from the guideline was detected, the clinician who booked the patient for assessment in the hot clinic was identified and notified to improve their practice. This monitoring program was a continuous program and was incorporated into normal commitments of on-call general surgery junior clinicians.

Data analysis

We hypothesized that the baseline compliance with the hot clinic criteria was 50%, and the target compliance in this study was 100%; therefore, it was estimated that a minimum number of 15 patients per cycle would be required to achieve 80% power with a 95% confidence level. Simple descriptive statistics were applied to present outcome data. Data were summarized with median and interquartile range (IQR) for continuous variables and frequencies/percentages for categorical variables. Differences between the baseline audit and re-audits were tested for statistical significance using the Mann-Whitney U test for continuous variables and the χ^2^ test for categorical variables. All statistical tests were two-tailed, and statistical significance was assumed at P < 0.05. The MedCalc 13.0 software (MedCalc Software Ltd., Ostend, Belgium) was used for statistical analyses.

## Results

**Figure 1:**
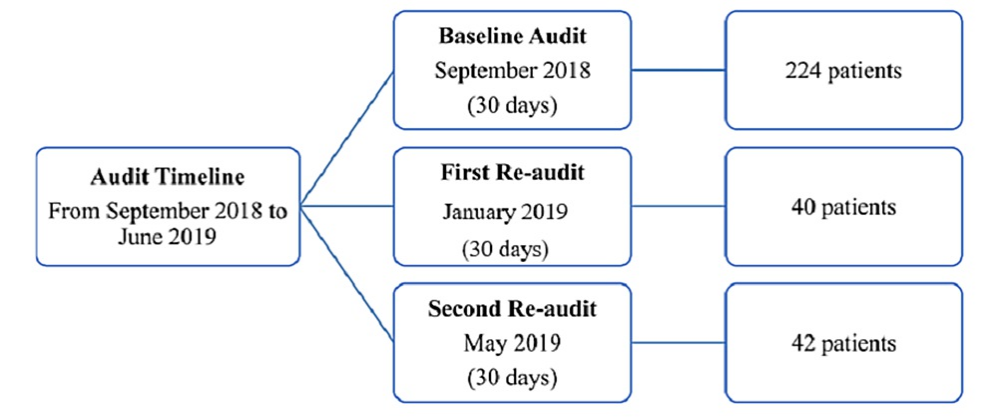
The flow chart for the audit

Overall, 306 patients were included in this audit: 224 patients in the baseline audit, 40 patients in the first re-audit, and 42 patients in the second re-audit. The study flow chart is shown in Figure 1.

Baseline audit

**Figure 2:**
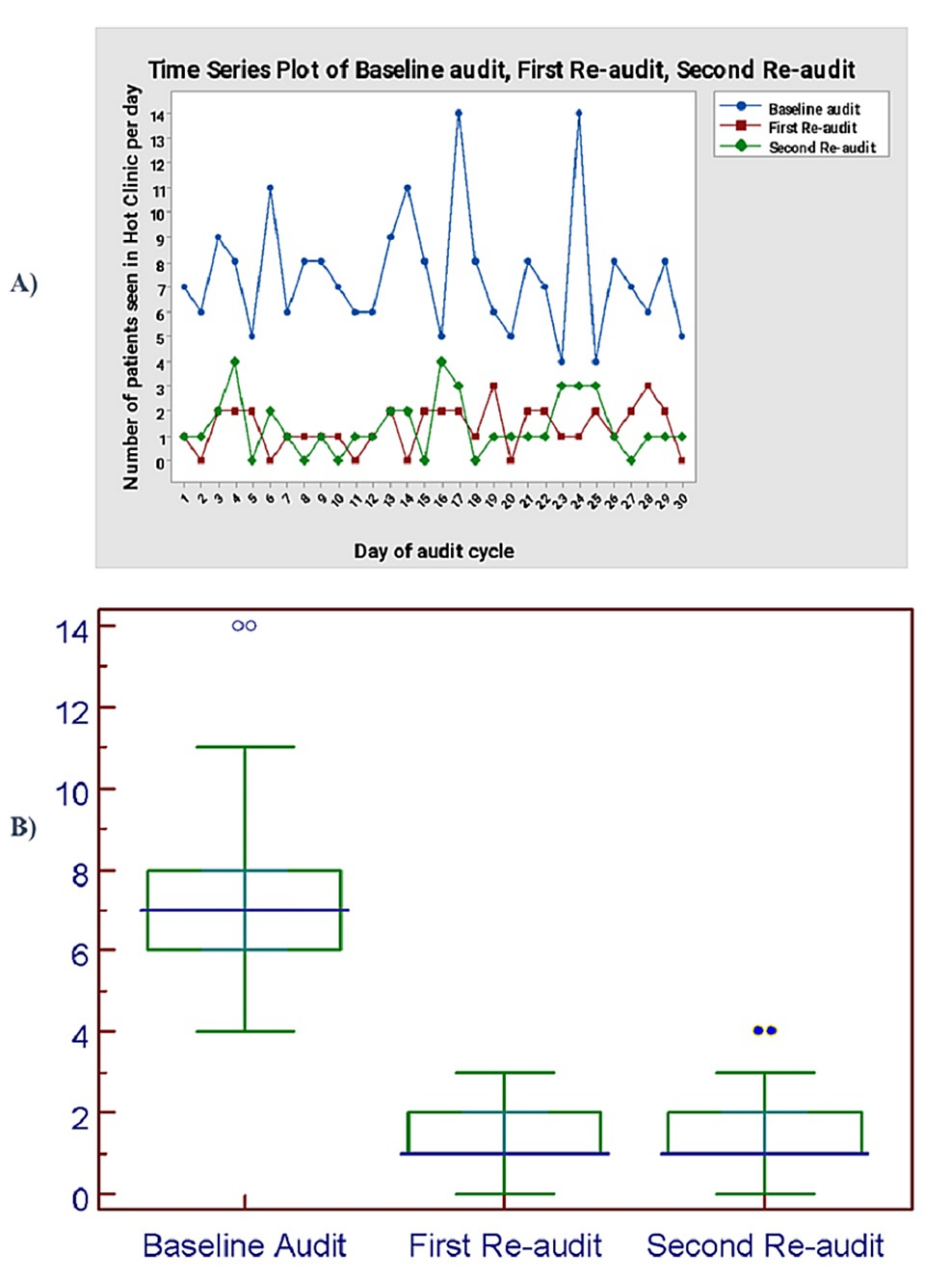
(A) Total number of patients seen in hot clinic per day. (B) Median number of patients seen in hot clinic per day.

**Figure 3:**
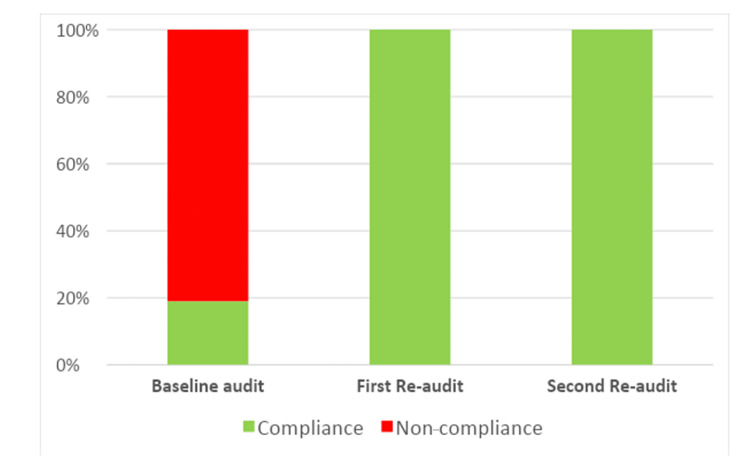
Compliance with standard 1: Had the patient been seen by the on-call registrar/SHO before the appointment? SHO: Senior house officer.

**Figure 4:**
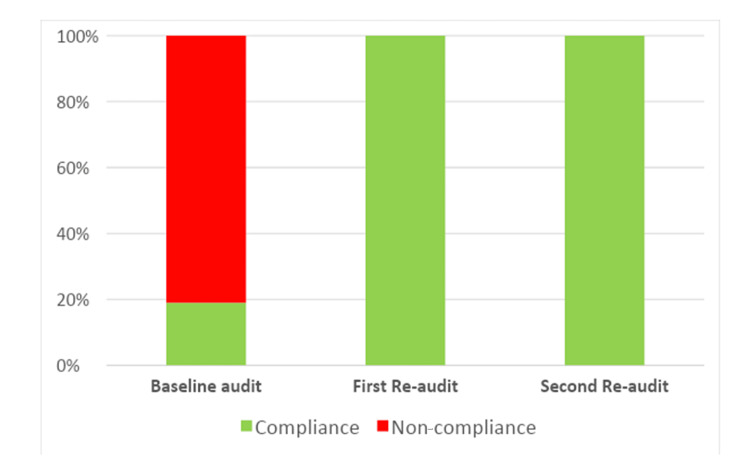
Compliance with standard 2: Did the patient meet the criteria for a hot clinic?

**Figure 5:**
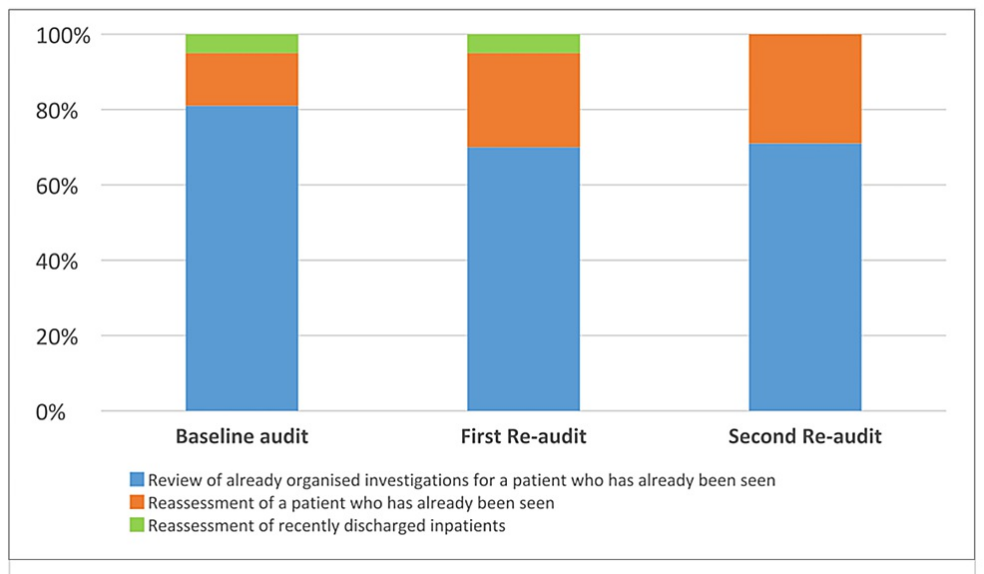
The reasons for hot clinic review among the patients who met the eligibility criteria

During the baseline audit, a total of 224 patients were seen in the general surgery hot clinic, and the median number of patients seen per day was 7 (6-8) (Figure 2). During the baseline audit, only 19% of patients had been seen by the on-call general surgery team prior to the hot clinic appointment (Figure 3). Among the 224 patients reviewed, only 19% met the eligibility criteria for review in a hot clinic (Figure 4). The reasons for the hot clinic review among the patients who met the eligibility criteria for ambulatory care during the baseline audit are shown in Figure 5.

First re-audit

After the implementation of the guideline, the total number of patients seen in the general surgery hot clinic decreased to 40 patients during the first re-audit period, and the median number of patients seen per day decreased to 1 (1-2) (Figure 2). There was a significant difference in the median number of patients seen per day between the baseline audit and the first re-audit (P < 0.0001). During the first re-audit, the proportion of the patients that had been seen by the on-call general surgery team prior to the hot clinic appointment improved to 100% (P < 0.0001) (Figure 3). Moreover, the proportion of the patients that met the eligibility criteria for review in the hot clinic improved to 100% (P < 0.0001) (Figure 4). The reasons for the hot clinic review among the patients who met the eligibility criteria for ambulatory care during the first re-audit are shown in Figure 5.

Second re-audit

During the second re-audit, the total number of patients seen in the general surgery hot clinic remained as low as 42 patients, and the median number of patients seen per day remained at 1 (1-2) (Figure 2). There was a significant difference in the median number of patients seen per day between the baseline audit and the second re-audit (P < 0.0001). During the second re-audit, the proportion of the patients that had been seen by the on-call general surgery team prior to the hot clinic appointment remained at 100% (P < 0.0001) (Figure 3). Moreover, the proportion of the patients that met the eligibility criteria for review in the hot clinic remained at 100% (P < 0.0001) (Figure 4). The reasons for the hot clinic review among the patients who met the eligibility criteria for ambulatory care during the second re-audit are shown in Figure 5.

## Discussion

A General surgery hot clinic is designed for the assessment and management of acute general surgical patients in ambulatory settings to avoid unnecessary hospital admissions. Overcrowding in a hot clinic is a major issue in many general surgical settings, and it is thought to be due to the lack of specific criteria-based guideline for identifying eligible patients for ambulatory care. We aimed to perform a prospective baseline audit to assess how many patients are seen in general surgery hot clinics on average and what proportion of hot clinic patients meet the criteria for ambulatory care. We also aimed to implement a specific criteria-based guideline and monitoring program to improve compliance with the ambulatory care criteria. The results of the baseline audit showed that compliance with hot clinic criteria was very poor. The implementation of a specific criteria-based guideline for the hot clinic significantly decreased the number of patients attending the hot clinic and improved compliance with ambulatory care criteria.

The existence of a criteria-based guideline for identifying eligible patients for ambulatory care would allow the on-call general surgery clinician to distinguish between the patients who can be managed as ambulatory patients and those who require to be managed as inpatient or outpatient. Moreover, it would help to improve the overcrowding in the hot clinic. Overcrowding results in reduced efficiency of ambulatory care and patient satisfaction. A criteria-based guideline prevents the on-call general surgery clinician from booking ineligible patients in the hot clinic or using the hot clinic as an excuse for not reviewing a referred patient on the same day as a referral.

As an absolute requirement for a patient to be seen in a hot clinic, the patient must have been seen and assessed by the on-call general surgery team prior to being booked in the hot clinic regardless of the source and time of referral. After the initial review by the on-call general surgery team, the patient can be booked in a hot clinic only for one of the following reasons: a review of already organized investigations for a patient who has already been seen, reassessment of a patient who has already been seen, and reassessment of a recently discharged inpatient. As demonstrated in this study, the above criteria would help to reduce the inappropriate referrals to the hot clinic and would help to improve the overcrowding in the hot clinic significantly, allowing the on-call general surgery team to run the hot clinic more efficiently.

The reported outcomes of this study should be viewed and interpreted in the context of inherent limitations. This audit was performed in a single general surgery setting, which affects the generalizability of our findings as different general surgery settings run their hot clinics differently, and some may have a team different from their on-call team responsible for hot clinic patients. Nevertheless, the criteria-based guideline for the hot clinic suggested in this study can be used in any general surgery setting, regardless of the aforementioned differences. Apart from the aforementioned limitations, adequate statistical power, prospective nature of this study, and consistent results during two re-audit cycles would make the results of the current study reliable with a low risk of type I or type II errors.

## Conclusions

A criteria-based hot clinic guideline suggested in this study improved compliance with the general surgery ambulatory care standards, the efficiency of the general surgery hot clinic, and overcrowding in the general surgery hot clinic. A continuous monitoring program led by an on-call junior general surgery doctor helped to maintain the aforementioned improvements.
